# Supplementary material for: The timescale and direction of influence of a third inferior alternative in human value-learning
Source: Commun Psychol. 2025 Apr 5;3:56. doi: 10.1038/s44271-025-00229-2 (PMC11972167; doi:10.1038/s44271-025-00229-2)
Supplement: Supplementary file 2 — Supplemental Material [file 44271_2025_229_MOESM2_ESM.pdf]

Supplemental Information of "The timescale and direction of influence of a third inferior alternative in human value-learning"

Maryam Tohidi-Moghaddam, Konstantinos Tsetsos

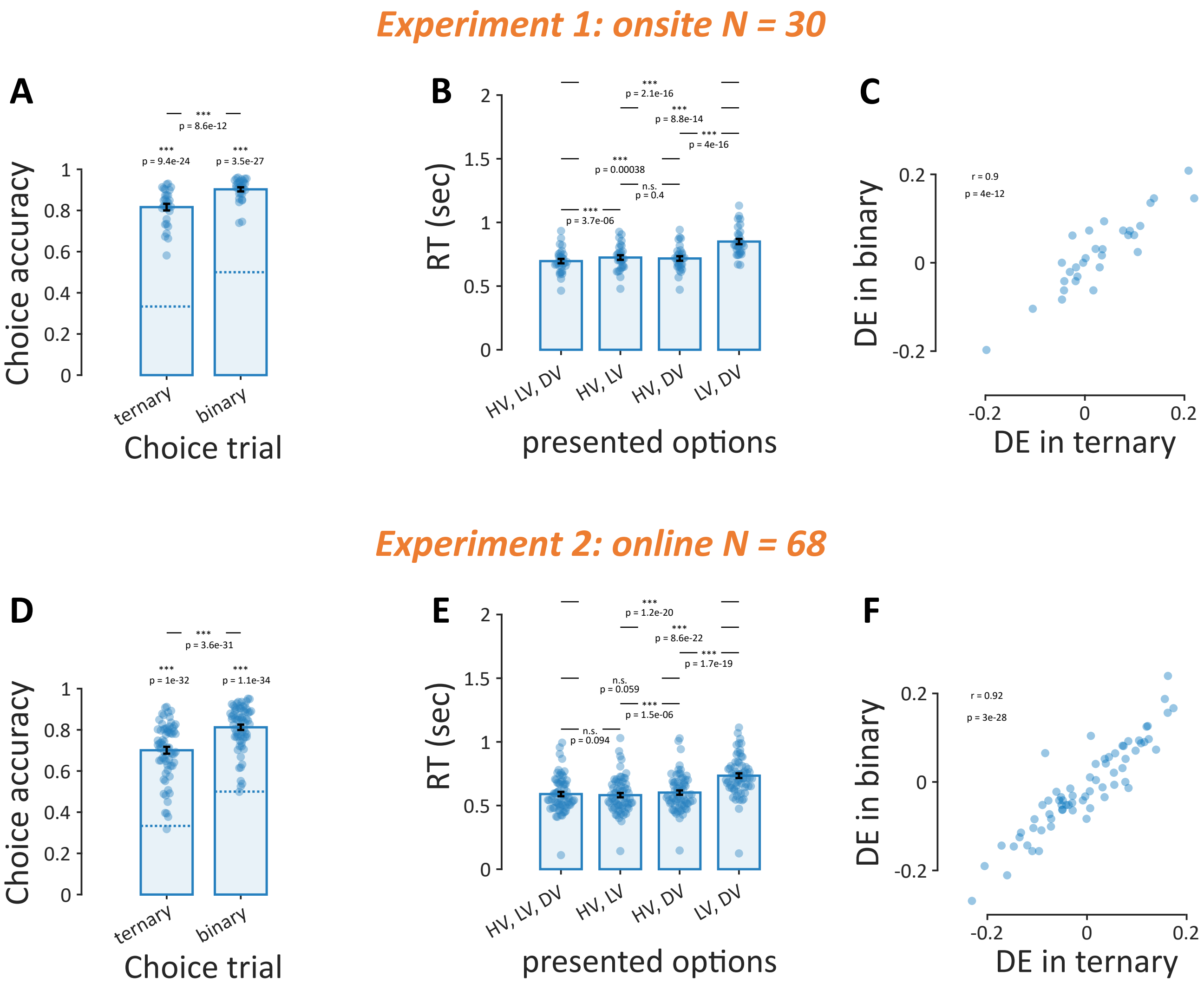

Figure S1. Complementary behavioural data from each experiment. (A, D) Choice accuracy of ternary and binary trials pooled across both "Feedback" and "No-Feedback" were significantly higher than chance. **(B, E)** The reaction time of participants' choices (both correct and incorrect choices) illustrated for each trial type. The RT in ternary trials is significantly lower than any binary trials. Within binary trial types, "LV, DV" pair has the significant higher RT than the other two binary pairs. **(C, F)** The correlation between the distractor effect in ternary and binary trials. The error bars are standard errors of the mean across participants and the dots indicate individual participants.

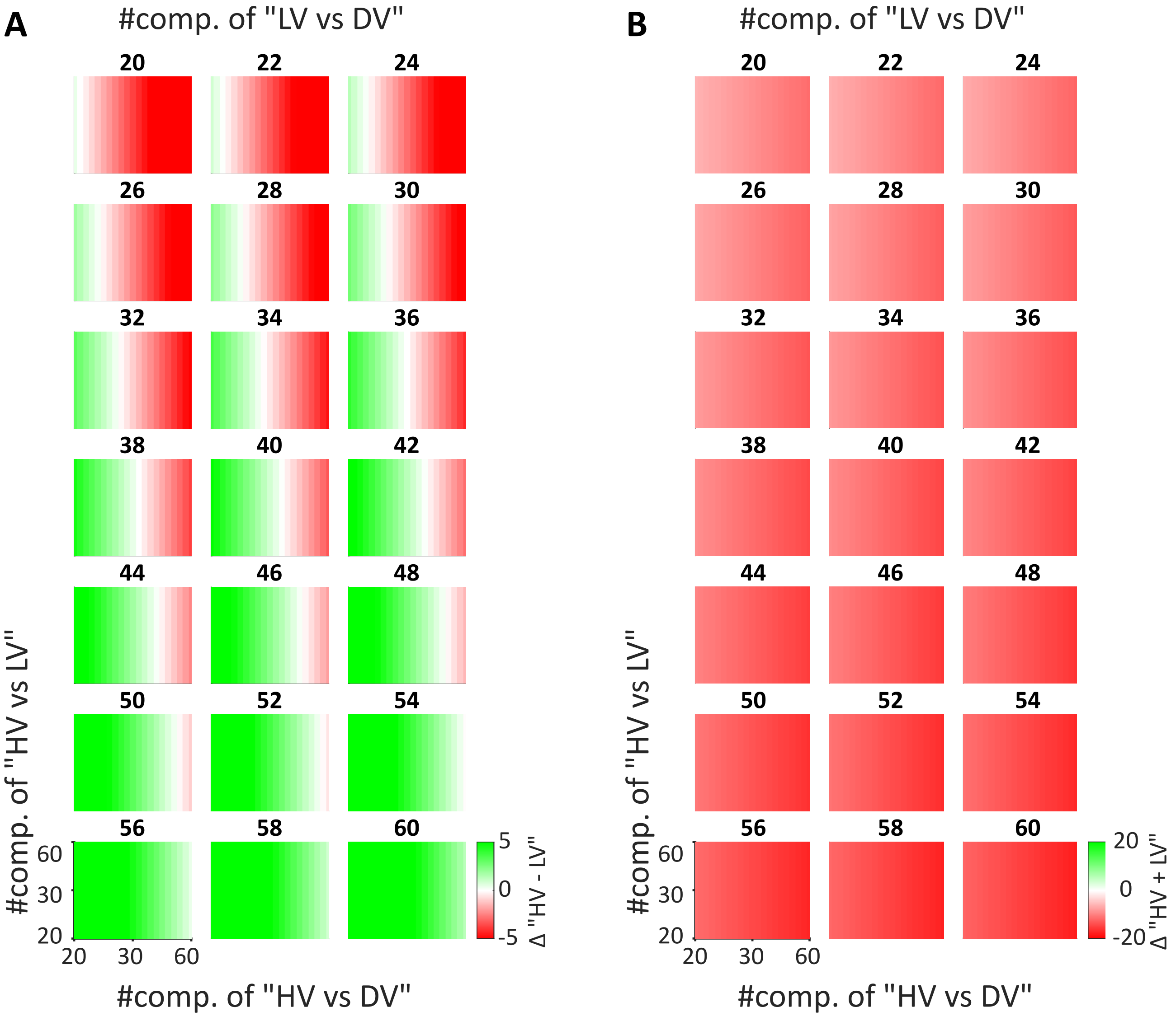

Figure S2. RB model simulation. (A) Delta of *"HV - LV"* as the function of the number of binary comparisons. (B) Delta of *"HV + LV"* as the function of the number of binary comparisons.

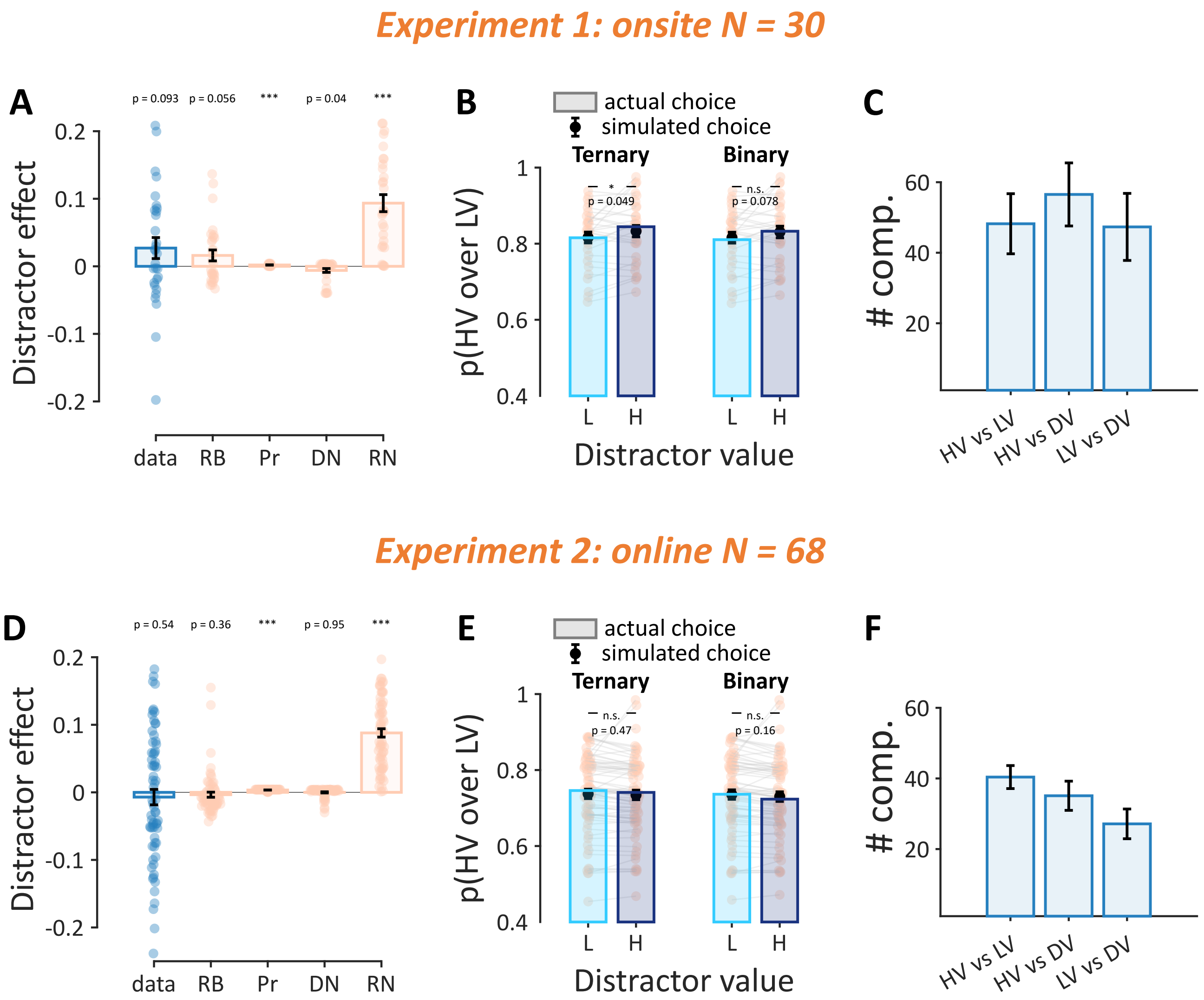

Figure S3. Model predictions. (A, D) Distractor effect comparison between the actual data and simulated data using the best-fitted parameters. **(B, E)** The simulated relative choice of the RB superimposed on the actual relative choice. **(C, F)** The distribution of fitted *k* parameters in each experiment (1-way ANOVA in Experiment 1: $F(2, 87) = .32$, $p = .73$, partial $\eta^2 = 0.01$, 95% CI [0.00, 0.05]; and Experiment 2: $F(2, 201) = 2.94$, $p = .06$, partial $\eta^2 = 0.03$, 95% CI [0.00, 0.05]). In all panels, the error bars are standard errors of the mean across participants, and the colored dots indicate the individual participant calculated using the best-fitted parameters of each model.

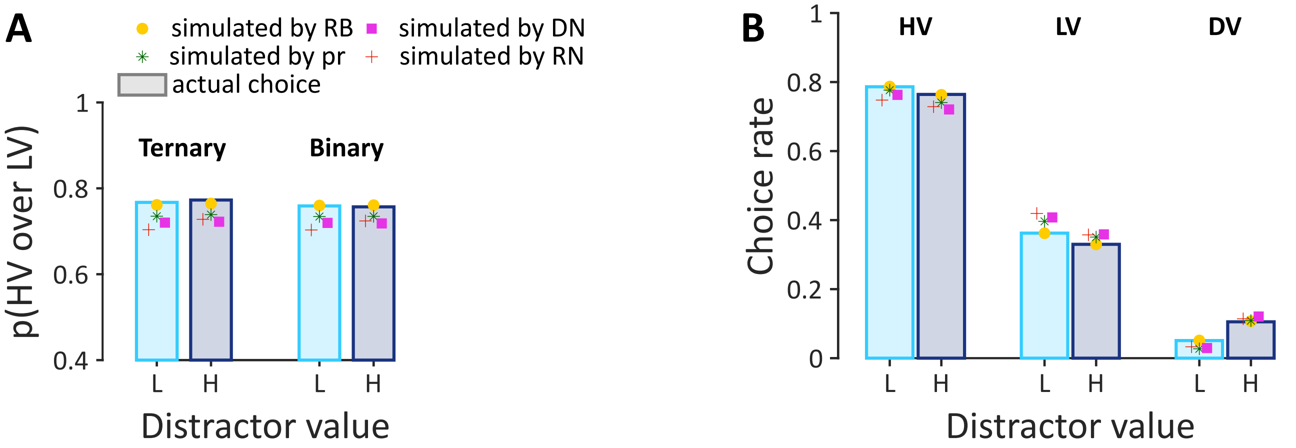

Figure S4. Model predictions. (A) The simulated relative probability of HV over LV, and **(B)** The simulated choice rate of each model superimposed on the actual data. In all panels, the simulated data points were calculated using the best-fitted parameters of each model, and the error bars are standard errors of the mean across participants.
